# *Schistosoma mansoni* Stomatin Like Protein-2 Is Located in the Tegument and Induces Partial Protection against Challenge Infection

**DOI:** 10.1371/journal.pntd.0000597

**Published:** 2010-02-09

**Authors:** Leonardo P. Farias, Fernanda C. Cardoso, Patricia A. Miyasato, Bogar O. Montoya, Cibele A. Tararam, Henrique K. Roffato, Toshie Kawano, Andrea Gazzinelli, Rodrigo Correa-Oliveira, Patricia S. Coulson, R. Alan Wilson, Sérgio C. Oliveira, Luciana C. C. Leite

**Affiliations:** 1 Centro de Biotecnologia, Instituto Butantan, São Paulo, SP, Brazil; 2 Departmento de Bioquímica e Imunologia, Instituto de Ciências Biológicas, Universidade Federal de Minas Gerais, Belo Horizonte, MG, Brazil; 3 Escola de Enfermagem, Universidade Federal de Minas Gerais, Belo Horizonte, MG, Brazil; 4 Laboratório de Imunologia e Biologia Molecular, Centro de Pesquisas René Rachou (CPqRR), Fiocruz, Belo Horizonte, MG, Brazil; 5 Department of Biology, University of York, York, United Kingdom; George Washington University, United States of America

## Abstract

**Background:**

Schistosomiasis affects more than 200 million individuals worldwide, with a further 650 million living at risk of infection, constituting a severe health problem in developing countries. Even though an effective treatment exists, it does not prevent re-infection, and the development of an effective vaccine still remains the most desirable means of control for this disease.

**Methodology/Principal Findings:**

Herein, we report the cloning and characterization of a *S. mansoni* Stomatin-like protein 2 (SmStoLP-2). *In silico* analysis predicts three putative sites for palmitoylation (Cys11, Cys61 and Cys330), which could contribute to protein membrane association; and a putative mitochondrial targeting sequence, similar to that described for human Stomatin-like protein 2 (HuSLP-2). The protein was detected by Western blot with comparable levels in all stages across the parasite life cycle. Fractionation by differential centrifugation of schistosome tegument suggested that SmStoLP-2 displays a dual targeting to the tegument membranes and mitochondria; additionally, immunolocalization experiments confirm its localization in the tegument of the adult worms and, more importantly, in 7-day-old schistosomula. Analysis of the antibody isotype profile to rSmStoLP-2 in the sera of patients living in endemic areas for schistosomiasis revealed that IgG1, IgG2, IgG3 and IgA antibodies were predominant in sera of individuals resistant to reinfection as compared to those susceptible. Next, immunization of mice with rSmStoLP-2 engendered a 30%–32% reduction in adult worm burden. Protective immunity in mice was associated with specific anti-rSmStoLP-2 IgG1 and IgG2a antibodies and elevated production of IFN-γ and TNF-α, while no IL-4 production was detected, suggesting a Th1-predominant immune response.

**Conclusions/Significance:**

Data presented here demonstrate that SmStoLP-2 is a novel tegument protein located in the host-parasite interface. It is recognized by different subclasses of antibodies in patients resistant and susceptible to reinfection and, based on the data from murine studies, shows protective potential against schistosomiasis. These results indicate that SmStoLP-2 could be useful in a combination vaccine.

## Author Summary

Schistosomiasis is a parasitic disease causing serious chronic morbidity in tropical countries. Together with the publication of the transcriptome database, a series of new vaccine candidates were proposed based on their functional classification. However, the prediction of vaccine candidates from sequence information or even by proteomics or microarrays data is somewhat speculative and there remains the considerable task of functional analysis of each new gene/protein. In this study, we present the characterization of one of these molecules, a stomatin like protein 2 (SmStoLP-2). Sequence analysis predicts signals that could contribute to protein membrane association and mitochondrial targeting, which was confirmed by differential extractions of schistosome tegument membranes and mitochondria. Additionally, confocal microscope analysis showed SmStoLP-2 present in the tegument of 7-day-old schistosomula and adult worms. Studies in patients living in endemic areas for schistosomiasis revealed high levels of IgG1, IgG2, IgG3 and IgA anti-SmStoLP-2 antibodies in individuals resistant to reinfection. Recombinant SmStoLP-2 protein, when used as vaccine, induced significant levels of protection in mice. This reduction in worm burden was associated with a typical Th1-type immune response. These results indicate that SmStoLP-2 could be useful in association with other antigens for the composition of a vaccine against schistosomiasis.

## Introduction

Schistosomiasis is an important parasitic disease, caused by trematode worms of the genus *Schistosoma*; it affects approximately 200 million of individuals primarily in developing countries and an estimated additional 500 to 600 million are at risk. The digenetic blood fluke, *Schistosoma mansoni*, is one of the major causative agents [1]. Parasite eggs are trapped in the liver and intestine, where they induce granuloma formation and fibrosis, the main cause of morbidity and mortality in schistosomiasis [2]. Chemotherapy is an important control strategy against this parasitic disease [3]; however, it has not reduced the endemicity [4] and rapid reinfection demands frequent treatment [5]. Therefore, it is considered that an effective vaccine combined with chemotherapy would be an efficient control mechanism [6].

Until October 2003, schistosome research suffered from limited genomic information; this situation has changed significantly with the simultaneous publication of the *S. mansoni* and *S. japonicum* transcriptomes [7],[8]. These initiatives, together with the advent of entire *S. mansoni* genome sequencing, all boosted by advances in bioinformatics, have markedly changed the schistosome vaccine research field. Simultaneously with the publication of the transcriptome data, and its scrutiny for genes with functions that would indicate their surface exposure to allow interaction with the host immune system, a series of novel genes were suggested as potential vaccine candidates based on their functional classification by Gene Ontology [8]. One of these, stomatin, was assigned a role in lipid raft formation or receptor binding by Gene Ontology categorization. Actually, it is most similar to the sub-family Stomatin Like Protein 2 (SLP-2), of which the best characterized gene is the human ortholog [9]. The protein was also proposed as a schistosome drug target [8], since the human ortholog was described as interacting with anti-malarial drugs, participating in the transfer of the drug Mefloquine to the intracellular parasite via a pathway used for the uptake of exogenous phospholipids [10].

The SLP-2 was first identified in humans (HuSLP-2); it presents, like other stomatins (e.g. Stomatin, SLP-1 and SLP-3), a central Stomatin, Prohibitin, Flotillin, HflK/C (SPFH) domain that may mediate interactions with plasma and mitochondrial membranes [11]–[13]. HuSLP-2 is the first member of this family that lacks an N-terminal hydrophobic domain, displaying a mitochondrial targeting sequence in this region. Additionally, a palmitoylation centered on Cys29 could not be identified [9],[11]. The function of stomatins, including SLP-2, remains undetermined. In erythrocytes, it may link stomatin or other integral membrane proteins to the peripheral cytoskeleton, playing a role in the regulation of ion channel conductance or in the organization of sphingolipids and cholesterol-rich lipid rafts [9]. More recently, this gene has been investigated as a novel cancer-related gene over-expressed in certain kinds of human tumours [14],[15], and in the assembly of mechanosensation receptors [16]–[22]. Moreover, it has been proposed to function as a link between synapse-polarized mitochondria and T-cell receptor (TCR) signalosomes, contributing to modulate TCR signalling and T cell activation [23],[24].

In this work, we describe and characterize a novel *S. mansoni* stomatin like protein 2 (SmStoLP-2). Data obtained here establishes that SmStoLP-2 is present in the tegument of adult worms and schistosomula. In addition, we evaluated the reactivity of rSmStoLP-2 antigen against the sera from individuals living in endemic areas for schistosomiasis in Brazil, showing that the groups resistant and susceptible to reinfection showed different antibody profiles. We subsequently demonstrated the ability of anti-rSmStoLP-2 serum to inhibit penetration and migration of cercariae *in vivo*. Lastly, immunization of mice with rSmStoLP-2 induced a Th1-type of immune response and a significant reduction in worm burden upon challenge with cercariae.

## Materials and Methods

### Parasite maintenance

*Schistosoma mansoni* adult worms (BH strain) were obtained by perfusion of mice, 7–8 weeks after infection. Eggs, miracidia, cercariae, and schistosomula were obtained as previously described [8]. Cercaria number and viability were determined using a light microscope prior to infection.

### Ethics statement

This study was conducted according to the principles expressed in the Declaration of Helsinki. The study was approved by the Institutional Review Board of Fundação Oswaldo Cruz (0083/99-CEP/FIOCRUZ). All patients or their legal guardians provided written informed consent for the collection of samples and subsequent analysis.

All animals were handled in strict accordance with good animal practice as defined by Animals Use Ethics Committee of UFMG (Universidade Federal de Minas Gerais, Brazil) and Instituto Butantan (São Paulo, Brazil), and the study was conducted adhering to the institution's guidelines for animal husbandry.

### Study population

Peripheral blood was obtained from individuals with different genetic background living in three endemic areas for schistosomiasis (‘Melquiades’, ‘Caatinga do Moura’ and ‘Côrrego do Onça’, all in the state of Minas Gerais, Brazil). These individuals were classified in four groups according to their infection status and the selection of subjects was performed based only on the criteria for inclusion and exclusion of each group independent of previous knowledge of immune responses for each individual. Non-infected (NI) individuals are healthy people from non-endemic areas without any parasite infection or contact with contaminated water. One group was shown to be stool-negative after treatment with praziquantel and was classified as resistant to *S. mansoni* reinfection (RR) [25]. The water contact exposure was determined using previously described methods [26]–[28], objectively evaluated by observers and studied population had at least one contact daily. Individuals classified as susceptible to *S. mansoni* reinfection (SR) were shown to be stool-positive following treatment with praziquantel (40 mg/kg) (at 1 and 5 months). The sera from RR and SR groups were obtained six months after praziquantel treatment and these individuals were examined for *S. mansoni* infections using the Kato–Katz technique before treatment and one, 6 and 12 months after treatment to check for reinfection rates [25]. Individuals grouped as infected (INF) showed stool-positive examination and no treatment history (never received anti-helminthic treatment, as determined by survey). These infected patients had infection levels that varied from 48 to 224 epg (egg counts per gram of feces). For each time point, three independent (consecutive days) stool samples were taken and two slides were prepared from each sample. These patients or their legal guardians gave informed consent after explanation of the protocol that had been previously approved by the Ethical Committee of Fundação Oswaldo Cruz. Details regarding sex and age of the individuals included in this study are described in Table S1.

### Cloning and molecular characterization of SmStoLP-2

Total RNA was isolated from adult worms (1 g) using TRIzol reagent (Invitrogen), followed by mRNA purification with oligo (dT)-cellulose columns according to the manufacturer's instructions (Amersham Biosciences). The SuperScript™ plasmid system for cDNA synthesis and cloning (Invitrogen) was used for cDNA library construction following the manufacturer's protocol. The cDNA fragments were directionally ligated into the SalI/NotI cloning sites of the pSPORT1 vector and transformed into competent *Escherichia coli* DH5α.

Specific oligonucleotides were designed using the EST assembly partial sequence from the São Paulo Transcriptome data (SmAE 606856.1, http://bioinfo.iq.usp.br/schisto6/) together with an EST from TIGR (BF936634). The 5′ and 3′ oligonucleotides, CACCATGATTCGTAGTATCATTGG and CTATTCTTGTTTATCGCTATC, were used in a PCR reaction to amplify the complete open reading frame of SmStoLP-2 from a cDNA library made from adult worms. The PCR reaction was performed using Platinum *Pfx* enzyme (Invitrogen), and initiated with one cycle of 5 min at 94°C, followed by 30 cycles of 30 s at 94°C, 1 min at 55°C, and 3 min at 68°C. PCR products were purified from agarose gel electrophoresis, cloned into pENTR/D TOPO cloning vector (Invitrogen), and sequenced to confirm its identity.

### Phylogenetic and sequence analysis

Blast and PSI-Blast searches against the non-redundant protein sequence database, using SmStoLP-2 as a query, were used to identify orthologs of SmStoLP-2. Additionally, we searched the *S. mansoni* genome (GeneDB, http://www.genedb.org/genedb/smansoni/) for proteins with Pfam SPFH/Band 7 domains. Post-translational modification prediction: the signal peptide prediction was performed using the SignalP 3.0 server (http://www.cbs.dtu.dk/services/SignalP/), transmembrane helices were analyzed by TMHMM version 2.0 (http://www.cbs.dtu.dk/services/TMHMM-2.0/), palmitoylation sites were predicted by CSS-Palm (http://csspalm.biocuckoo.org/1.0/index.php) [29], and mitochondrial targeting sequence as predicted by the MitoProt program (http://ihg2.helmholtz-muenchen.de/ihg/mitoprot.html). Molecular weight (MW) and isoelectric point (pI) were calculated with the Compute pI/Mw tool (http://www.expasy.ch/tools/pi_tool.html).

For phylogenetic analyses, alignments of protein sequences were performed using the ClustalX 1.83 software. The tree was constructed using Clustal with the Neighbour Joining method, excluding positions with gaps. The numbers represent the confidence of the branches assigned by bootstrap (in 1000 samplings). The TreeView program (http://taxonomy.zoology.gla.ac.uk/rod/treeview.html) was used to visualize the tree.

### Expression of recombinant protein and polyclonal antibody production

To produce a recombinant SmStoLP-2, the full-length cDNA sequence was directionally cloned by recombination into pDEST17 (to produce a protein that contains an N-terminal hexahistidine tag) and transformed into BL21 (DE3) (Invitrogen). For protein expression, the transformed cells were grown in 600 ml LB plus ampicilin (OD_600_ = 0.6). Isopropyl-β-D-thiogalactopyranoside (IPTG) was added to the culture to a final concentration of 1 mM, and cells were incubated for 3–4 h at 37°C. Cells were harvested by centrifugation and resuspended in 50 ml of lysis buffer (50 mM sodium phosphate pH 8.3, 0.3 M NaCl). The cell suspension was passed twice (1500 psi) through a French press and the crude homogenate was centrifuged at 20,000×*g* for 40 min. The pelleted inclusion bodies were washed twice with wash buffer (lysis buffer, 2% Triton X-100, 2 M urea) and finally resuspended in solubilization buffer (lysis buffer, 5 mM beta-mercaptoethanol, 20 mM imidazole, 8 M urea). The recombinant protein was refolded from the inclusion bodies by diluting 100-fold into equilibration buffer (solubilization buffer without urea).

The recombinant protein was then purified by metal affinity chromatography using the Akta Prime system (Amersham Biosciences) under native conditions. Briefly, the sample was loaded onto a Ni^2+^-NTA column (5 ml bed volume) pre-equilibrated with the same buffer. The column was washed with 10 bed volumes of the equilibration buffer and then eluted with 20–500 mM imidazole linear gradient. The main peak was pooled and the protein purity of fractions was assessed using sodium dodecyl sulfate polyacrylamide gel electrophoresis (SDS-PAGE). Further, the elution buffer was exchanged with Phosphate Buffer Saline pH 7.4 (PBS) before use of this protein.

Polyclonal rat serum was produced against preparations of recombinant SmStoLP-2. Rodents were inoculated four times, at 21-day intervals with 100 µg of protein mixed with TiterMax adjuvant (CytRx Corporation; first dose) or PBS (in subsequent doses). Fifteen days after the last inoculation, rodents were exsanguinated. The sera were used at a dilution of 1∶10,000 (v∶v) in Western blots and 1∶100 in indirect immunofluorescence assays.

### Circular dichroism (CD) measurements

CD measurements were carried out on a Jasco J-810 Spectropolarimeter at 20°C equipped with a Peltier unit for temperature control. Far-UV CD spectrum was acquired using a 1 mm path length cell at 0.5 nm intervals over the wavelength range from 190 to 260 nm. Five scans were averaged for each sample and subtracted from the blank average spectra. The protein concentration was kept at 10 µM in 10 mM sodium phosphate buffer pH 7.4.

### Protein expression profile

Total parasite extracts from eggs, miracidia, cercariae, 10-day old schistosomula and adult worms of *S. mansoni* were prepared in 40 mM Tris, pH 7.4, 2% SDS plus protease inhibitor cocktail (Sigma) through sonication (4 cycles of 2 min, with pulses of 0.75 s, 40% amplitude). The samples were centrifuged at 20,000×*g* for 30 min at 4°C and the supernatant was quantified and used for assays. The soluble fraction of adult worms and schistosomula was obtained in a similar way, with the exception of 2% SDS in the sonication buffer. After centrifugation at 20,000×*g* for 30 min at 4°C, the supernatant was recovered, and the insoluble pellet was sonicated in the presence of 2% SDS, which after centrifugation at 20,000×*g* for 30 min at 4°C, originated the so-called insoluble fraction. Their protein concentrations were determined with a RC DC Protein Assay Kit (Bio-Rad, CA, USA). Samples of purified rSmStoLP-2 and extracts (20 µg) were submitted to SDS-PAGE. The gel was electroblotted onto a PVDF membrane, which was blocked with 0.02 M Tris (pH 7.5) and 0.3% Tween 20 containing 5% dry milk for 16 h at 4°C. Subsequently, the membrane was incubated in a 1∶10,000 dilution with primary antibody in blocking buffer plus 150 mM NaCl for 3 h at room temperature. After three washes using Tris 10 mM (pH 7.5), the membrane was incubated in a 1∶4000 dilution with secondary goat anti-rat IgG conjugated to horseradish peroxidase (HRP) (Pierce) for 1 h and after three washes using Tris 10 mM (pH 7.5), the membrane was treated with ECL plus (GE) reagent according to manufacturer's instructions.

### Tegument removal, differential extraction and fractionation: Surface membranes and mitochondrial enrichment

The sample used in this experiment was kindly provided by Dr. Simon Braschi (University of York, England, UK). Briefly, the tegument was removed by a freeze/thaw method and surface membranes enriched by sucrose-gradient centrifugation as previously described [30],[31], generating a gradient pellet. Proteins were sequentially extracted from the gradient pellet using a three-step process with reagents of increasing solubilizing power as follows: Extract 1 (soluble proteins): 40 mM Tris, pH 7.4; Extract 2 (non-covalent, but firmly bound proteins): 5 M urea (BDH, VWR International, Dorset, UK), 2 M thiourea (BDH) in 40 mM Tris, pH 7.4 (Extraction Buffer 2; EB2); Extract 3 (GPI-anchored and single membrane spanners): EB2 plus 4% CHAPS (Sigma) and 2% N-decyl-N, N-dimethyl-3-ammonio-1-propane sulphate (SB 3–10; Sigma), pH 7.4; Final pellet (multispanning membrane proteins): solubilized with 40 mM Tris, pH 7.4 plus 2% SDS.

Mitochondrial enriched fraction was prepared from adult worm tegument in isotonic mitochondrial buffer (MB) (210 mM mannitol, 70 mM sucrose, 1 mM EDTA, 10 mM HEPES pH 7.5) supplemented with complete protease inhibitor cocktail (Sigma). The tegument membranes were obtained by centrifugation at 100×*g* for 30 min at 4°C. The resulting supernatant was centrifuged at 10,000×*g* for 10 min at 4°C to purify the mitochondrial fraction. The resulting pellets were ressuspended in 40 mM Tris, pH 7.4 plus 2% SDS. The protein concentration was estimated by the method of Lowry with a RC DC Protein Assay Kit (Bio-Rad, CA, USA). Anti-Mitofusin-1 antibody (Mfn1 (H-65) (Santa Cruz Biotechnology) (1∶200 dilution) was used as a mitochondrial tracker in Western blot experiments followed by incubation with secondary goat anti-rabbit IgG conjugated to HRP (Sigma).

### Indirect immunofluorescence and confocal microscopy

Freshly perfused worms were embedded in OCT medium in a pre-cooled beaker of isopentene, frozen in liquid N_2_. Eight-micrometer cryostat adult worm sections were adhered to silanized glass slides (DakoCytomation) and fixed in acetone for 30 min at −20°C before blocking with PNT (PBS 1x, 10% Naive rabbit serum and 0.1% Tween 20) for 4 h at room temperature. They were then incubated with anti-rSmStoLP-2 antisera diluted 1∶100 in PNT for 2 h at room temperature. After washing five times with PBS 0.1% Tween 20, pH 7.4 (PBS-T), an Alexa Fluor 488 conjugated anti-rat IgG 1∶200 (v∶v), 20 mM DAPI (4′, 6-diamidino-2-phenylindole dihydrochloride, Molecular Probes) to visualize nuclei, and 0.1 µg/ml phalloidin rhodamine (Molecular Probes) to stain actin microfilaments, were added to PNT solution, and the samples incubated for 1 h at room temperature. Sections were washed five times with PBS-T, and then mounted in Fluorescent Mounting Medium (DakoCytomation). In order to label the whole parasite, 7-day cultured schistosomula were fixed in 4% paraformaldehyde in phosphate-buffered saline (PBS) for 1 h on ice, washed with PBS and kept at 4°C until use, the hybridisation conditions were the same used for adult worm sections. Rat pre-immune sera were used as negative control. Images were acquired in a Zeiss LSM 510 Meta confocal system, attached to a Zeiss Axiovert 100 microscope using a LD-Achroplan 20x/0.4 or C-Apochromatic 63x/1.2 water immersion objectives with differential interference contrast.

### Measurement of human humoral response to SmStoLP-2

Sera of schistosomiasis patients living in endemic areas in Brazil were tested by ELISA as previously described [32]–[34] to measure the levels of immunoglobulin isoytpes to rSmStoLP-2 protein. For this assay, 96 well flat-bottom microtiter plates (Nunc) were coated overnight at 4°C with 100 µl/well of rSmStoLP-2 at a concentration of 5 µg/ml in 0.1 M carbonate bicarbonate buffer (pH 9.6). The plates were then blocked with 10% bovine fetal serum in PBS (pH 7.4) for 2 h at room temperature. Subsequently, the plates were washed three times with PBS plus 0.05% Tween-20 (PBS-T). Serum samples diluted 1∶50 (IgG) and 1∶40 (IgA) in PBS-T (100 µl/well) were added in duplicate and the plates incubated for 1 h at room temperature. Peroxidase-labelled anti-human IgG and anti-human IgA (Sigma) was added at dilutions of 1∶10,000 and 1∶1000 (100 µl/well), respectively. After 1 h at 37°C, the plates were washed and orthophenyl-diaminobenzidine plus 0.05% hydrogen peroxide in phosphate citrate buffer (pH 5) was added (100 µl/well). This mixture was than incubated for 15 min at room temperature, and the reaction was stopped by addition of 5% H_2_SO_4_ (50 µl/well). Absorbance was read at 492 nm using a microplate reader (Bio-Rad, Hercules. CA, USA). To measure IgG subclasses, the previous protocol was modified. The serum dilution was changed based on the isotype to be detected. Serum samples diluted 1∶30 (IgG1), 1∶5 (IgG2 or IgG4) and 1∶80 (IgG3) were added to the plates and incubated for 2 h at 37°C, as previously described [32]. After washing, peroxidase labelled mouse anti-human antibody was added in each well at concentrations of 1∶1000 (IgG1, IgG3, IgG2 or IgG4), and the plates were incubated for 16 h at 4°C. The subsequent steps were identical to those described for the other isotypes.

### Cercariae penetration and migration inhibition assay

Cercariae penetration and migration inhibition assays were adapted from a previously described method for *Necator americanus* [35]. Briefly, six aliquots of 100 µl, each containing 100 cercariae of *S. mansoni*, were incubated for 1 h at 37°C with 50 µl of sera from rats immunized with rSmStoLP-2 formulated with TiterMax. Sera from rats injected with saline was used as control. To eliminate any effect of complement, the sera were previously heated for 30 min at 56°C. After the 1h incubation period, 800 µl of pond water was added to the samples and the whole volume (950 µl) was then applied to the shaven abdomen of six anaesthetized mice (90 mg/Kg of Ketamine and 10 mg/Kg of Xylazine). The cercariae were allowed to penetrate by the ring method for 30 min at room temperature. Non-penetrating parasites were evaluated by counting those that remained on the surface of the skin, which were collected by removing the remaining liquid with a pipette and washing the skin twice with 1 ml of PBS. The mean value was considered the percentage of larval penetration inhibition by the antiserum. Six weeks after percutaneous penetration, 6 mice per group were sacrificed with a lethal dose of Ketamine/Xylazine solution. Perfusion fluid (Saline solution, 500 units/L of heparin) was pumped into the aorta artery, and perfused worms were collected from the hepatic portal vein. Adult male and female worms were counted using a stereomicroscope.

### Immunization of mice, challenge infections and parasite loads

Five to six week-old female C57BL/6 from the Universidade Federal de Minas Gerais (UFMG) animal facility, were supplied with food and water *ad libitum*. Groups of C57BL/6 mice were lightly anaesthetized (with 45 mg/kg of Ketamine and 10 mg/kg of Xylazine) and injected subcutaneously in the nape of the neck with 3 doses, at 15-day intervals, of 25 µg of protein mixed with Freund's Complete Adjuvant (Sigma; first dose) or Freund's Incomplete Adjuvant (in subsequent doses). In the control group, PBS with Freund's adjuvant was administered using the same immunization protocol. Challenge infections were performed 2 weeks following the final immunization. Mice were anaesthetized with 90 mg/Kg of Ketamine and 10 mg/Kg of Xylazine and exposed percutaneously to 100 cercariae by the ring method on their shaven abdomens. Six weeks after percutaneous challenge infections, 10 mice per group were sacrificed and perfused as described in the cercariae inhibition of penetration assay. The protection was calculated by comparing the number of worms recovered from each vaccinated group with its respective control group, in two independent experiments.

The livers were collected from the same animals fixed in 10% paraformaldehyde, processed for paraffin embedding and histopathological sections performed using microtome at 6–7 µm and stained in a slide with hematoxilin-eosin (HE). The number of granulomas was obtained from the liver sections using 10× objective in a microscope. The area from each liver section was calculated using capture in scanner followed by analysis in the KS300 software connected to a Carl Zeiss image analyzer, and the number of granulomas calculated by the area of the liver.

### Humoral response in mice immunized with rSmStoLP-2

Mice were bled from the retro orbital plexus and ELISA was performed to confirm the titer of specific anti-rSmStoLP-2 IgG, IgG1 and IgG2a in the serum of immunized animals. Briefly, 96 well flat-bottom microtiter plates (Nunc) were coated overnight at 4°C with 100 µl/well of rSmStoLP-2 at a concentration of 5 µg/ml in a 0.1 M carbonate bicarbonate buffer (pH 9.6). The plate was then blocked with bovine fetal serum 10% in PBS for 2 h at room temperature. Further, the plates were washed three times with PBS plus 0.05% Tween-20 (PBS-T). One hundred microliters of each serum diluted 1∶100 in PBS-T was added per well and incubated for 1 h at room temperature. Plate-bound antibody was detected by peroxidase-conjugated anti-mouse IgG, IgG1 and IgG2a (Southern Biotechnology) diluted in PBST 1∶10,000, 1∶5000 and 1∶2000, respectively. After 1 h at 37°C, the plate was washed and orthophenyl-diaminobenzidine plus 0.05% hydrogen peroxide in phosphate citrate buffer (pH 5) was added (100 µl/well). This mixture was then incubated for 30 min at room temperature, and the reaction was stopped by addition of 5% H_2_SO_4_ (50 µl/well). Absorbance was read at 492 nm using a microplate reader (Bio-Rad, Hercules, CA, USA). Animals that received PBS with Freund's adjuvant were used as negative control.

### ELISA detection of IFN-γ, IL-4, IL-10 and TNF-α in the supernatant of spleen cell cultures from mice immunized with rSmStoLP-2

Cytokine experiments were performed using splenocyte cultures from individual mice immunized with rSmStoLP-2 plus CFA/IFA (*n* = 5 for each group). Splenocytes were isolated from macerated spleens of individual mice 10 days after the third immunization and washed twice with sterile PBS. The cells were adjusted to 1×10^6^ cells per well in RPMI 1640 medium (Gibco, CA, USA) supplemented with 10% FBS, 100 U/ml penicillin G sodium, 100 µg/ml streptomycin sulfate, 250 ng/ml amphotericin B. Splenocytes were maintained in culture with medium alone or stimulated with rSmStoLP-2 (25 µg/ml) or concanavalin A (ConA) (5µg/ml) as previously described [36]. The 96-well plates (Nunc) were maintained in an incubator at 37°C with 5% CO_2_. For cytokine assays, polymyxin B (30 µg/ml) was added to the cultures and this treatment completely abrogated the cytokine response to LPS, as previously described [37]. Culture supernatants were collected after 48 h of rSmStoLP-2 stimulation for IL-4 and TNF-α analysis and 72 h of rSmStoLP-2 stimulation for IL-10 and IFN-γ. The assays for measurement of IL-4, IL-10, IFN-γ and TNF-α were performed using the Duoset ELISA kit (R&D Diagnostic) according to the manufacturer's recommendations.

### Statistical analysis

Student's *t*-test was used and the two-tailed *p*-value was calculated to compare experimental and control groups on challenge infections, antibody profiles and cytokine assays in mice. For the human humoral response against rSmStoLP-2, the Kruskal–Wallis test was used to evaluate the significance of the results of all groups compared to the non-infected (NI). The Mann–Whitney test was used to evaluate the significance of antibody measurements obtained between the groups resistant to *S. mansoni* reinfection (RR) *versus* the groups susceptible to reinfection (SR).

## Results

### Cloning and molecular characterization of *S. mansoni* StoLP-2

The full-length sequence of the *S. mansoni* cDNA encoding Stomatin like protein-2 was obtained by PCR from an adult worm cDNA library with specific oligonucleotides. The resulting full-length cDNA (GenBank accession EU531730) displays an ORF of 1077 bp, encoding a protein of 358 amino acids with a predicted molecular mass of approximately 39.5 kDa and an isoelectric point of 5.83. BlastP comparisons of the deduced *S. mansoni* protein sequence to GenBank showed that the best match (E-value = 5×10^−95^) was to *Danio rerio* hypothetical protein, with 58% identity and 81% similarity over 355 amino acids. The next best match was against an unknown *S. japonicum* protein (probably an incomplete SLP-2). This was followed by several other SLP-2 proteins including human (58% of identity), therefore we designated this gene as SmStoLP-2 (since there was already another gene named as Sm-SLP-2, although not related to Stomatin like proteins [38]).

**Figure 1:**
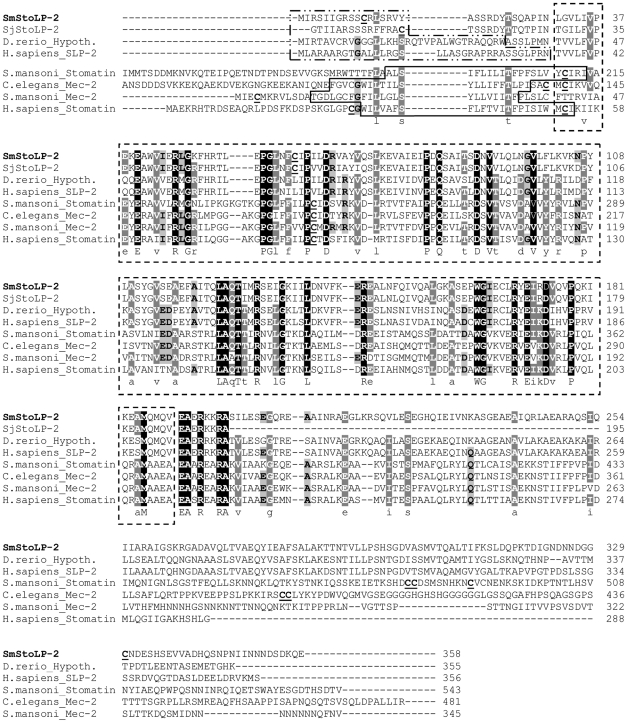
The complete protein sequence of SmStoLP-2 in relation to other members of the stomatin gene family. ClustalX alignment of the derived amino acid sequence of SmStoLP-2 (**EU531730**) with *S. japonicum* StoLP-2 (**AAX30477**), *D. rerio* Hypothetical (**NP_957325**), *H. sapiens* SLP-2 (**NP_038470**), *S. mansoni* stomatin excluding the first 120 amino acids (GeneDB accession no. Smp_162440), *C. elegans* Mec-2 excluding the first 50 amino acids (**NP_741797**), *S. mansoni* Mec-2 (GeneDB accession no. Smp_122810), *H. sapiens* stomatin (**NP_004090**), *H. sapiens* SLP-3 (**NP_660329**), *M. musculus* SLP-3 (**NP_694796**), *H. sapiens* SLP-1 (**NP_004800**) and *C. elegans* Unc-24 (**NP_501335**). Highlighted are the putative transmembrane domains as predicted by TMHMM (continuous box) absent in all SLP-2, the stomatin signature sequence (dashed box), mitochondrial targeting sequence as predicted by MitoProt II (dashed and dotted box), sites for palmitoylation (underlined). The regions with high identity and similarity between stomatin sequences are shown as black and gray columns, according to the Clustal X algorithm.

SmStoLP-2 contains the stomatin signature sequence (residues 31–189) (outlined by a dashed box in Figure 1), and is recognized as part of the Pfam SPFH/Band 7 family with an E-value of 1.2×10^−75^. Additionally, searching the *S. mansoni* genome (GeneDB) for proteins with Pfam SPFH/Band 7 domains, we found putative orthologues of *H. sapiens* stomatin (Band 7), and *C. elegans* Mec-2 (Figure 1). We identified a further five schistosome stomatin-related genes (data not shown).

Human stomatin (Band 7) may associate with membranes via a hairpin loop (continuous box) with both the N- and C- termini facing the cytoplasm (Figure 1). This domain is conserved among several members of the SPFH/Band 7 superfamily, such as *C. elegans* MEC-2 and *S. mansoni* Stomatin and Mec-2, but is absent in SLP-2 members (Figure 1). We further identify in all SLP-2 sequences putative signal peptides (ranging from 16 to 32 amino acids at the N-terminal region), which were predicted to be a mitochondrial targeting sequence (dashed - dotted box). Some previously recognized stomatin family members, such as human stomatin, have a consensus sequence for palmitoylation centered on Cys29 and Cys86 [39], which apparently increase the affinity of stomatin for the membrane. Further examining the distribution of potential post-translational modifications of SmStoLP-2, we found three putative sites for lipid modification (palmitoylation) centered on Cys11, Cys61 and Cys330 (Figure 1, underlined). Surprisingly, these palmitoylation sites were not detected in any other analysed member of the SLP-2 subfamily, except for the *S. japonicum* ortholog (data not shown).

Phylogenetic analysis of the SFPH superfamily confirmed that SmStoLP-2 is a member of the stomatin family, grouping it in a branch with other SLP-2s (Figure S1). Like other stomatins [13],[40],[41], it is distantly related to flotillin, prohibitin, and HflK/C. Two putative flotillin and two putative prohibitins genes were identified in the *S. mansoni* genome. As expected, and probably due to its prokaryotic origin, no orthologues of HflK/C were found (Figure S1).

### Production of recombinant SmStoLP-2

**Figure 2:**
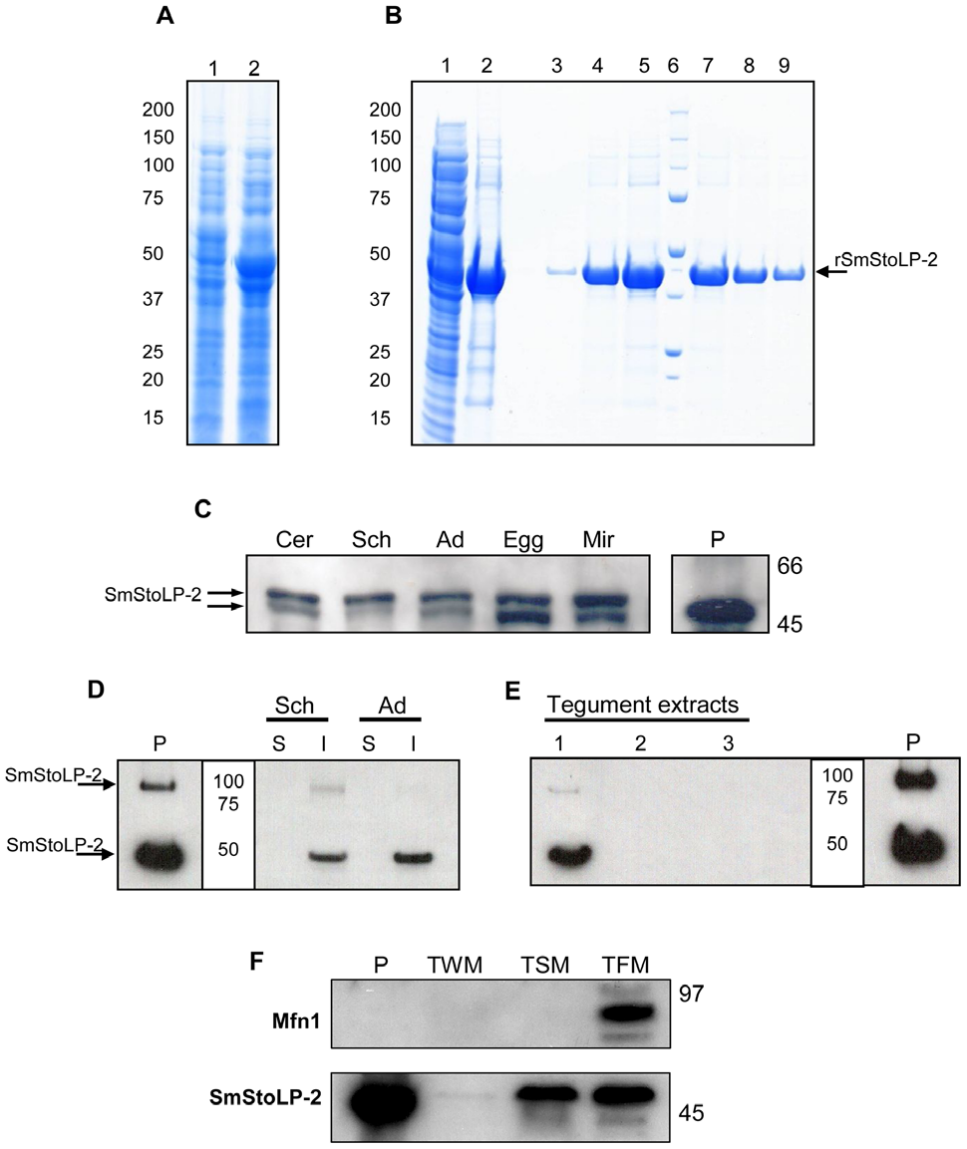
SDS-PAGE (4%–12%) analysis of cell extracts and fractions from *E. coli* (BL21DE3) transformed with the pDEST17-SmStoLP-2, and immunoblotting of protein extracts from *S. mansoni* stages and fractions using anti-rSmStoLP-2 polyclonal antibodies. (A) Lanes 1 and 2 represent a clone before and after induction with 1 mM IPTG, respectively; (B) Inclusion bodies were extracted with urea and denatured protein was refolded by dilution before being purified through Ni^2+^-charged column chromatography. Lanes 1 and 2 show the soluble fraction after lysis and the inclusion bodies after solubilization with 8 M urea, respectively. Lanes 3–5 and 7–9 show the fractions of rSmStoLP-2-6xHIS-tag fusion protein eluted after Ni^2+^ chromatography, Lane 6, MW ladder (kDa); (C) Immunoblotting of *S. mansoni* extracts from different stages using anti-rSmStoLP-2 polyclonal antibodies (20 µg of protein were loaded in each lane). Cer – cercariae, Sch – 7-day-old schistosomula, Ad – adult worms, Egg – eggs, Mir – miracidia. (D) Western blot of soluble (S) and insoluble (I) protein extracts of 10-day-old schistosomula (Sch) and adult worms (Ad); (E) Detection of SmStoLP-2 in the tegument of *S. mansoni* adult worms, (1) proteins soluble in urea and thiourea, (2) proteins soluble in urea, thiourea, CHAPS and SB 3–10, (3) proteins soluble in 2% SDS. (F) Dual targeting of SmStoLP-2 to tegumental membranes and tegumental mitochondria, TWM, tegument extract without surface membranes, TSM, tegument enriched in surface membranes and TFM, tegument fraction enriched in mitochondria (20 µg of protein were loaded in each lane), Mfn-1 – is the Mitofusin-1 mitochondrial marker. Arrows indicate the rSmStoLP-2 and the most reactive bands of native SmStoLP-2 detected in each experiment. Positions of molecular mass standards (kDa) are indicated on the right or in the center. Positive control (P) contains 50–60 ng of rSmStoLP-2.

*E. coli* transformed with pDEST17-SmStoLP-2 showed a band at 45 kDa when induced with IPTG, which is slightly higher than the expected molecular mass for rSmStoLP-2 (Figure 2A). The bacteria were lysed by a French Press and separated into soluble and insoluble fractions. The insoluble fraction (inclusion bodies) was shown to contain the majority of the recombinant protein (Figure 2B, lanes 1, 2). The inclusion bodies were extracted with 8 M urea, refolded by dilution and purified by affinity chromatography on nickel-charged columns through an imidazole linear gradient from 20 to 500 mM (Figure 2B, lanes 3–8). The fractions were pooled and dialyzed to remove imidazole, yielding 8.0 mg of rSmStoLP-2/L culture. Circular Dichroism spectra indicated that the rSmStoLP-2 contains a regular secondary structure, although the proportions of secondary structure elements (α-helix and β-sheet) were not calculated (data not shown).

### Analysis of protein expression across the life cycle stages

Extracts were prepared from cercariae, schistosomula, adult worms, eggs and miracidia stages of *S. mansoni* and subjected to immunoblotting with rat anti-rSmStoLP-2 serum, showing comparable levels of expression in all stages across the parasite life cycle. Native SmStoLP-2 observed in schistosome extracts, migrates with a molecular mass higher than that predicted, which was comparable to rSmStoLP-2 (∼49 kDa) (Figure 2C). It is not known whether an additional smaller band (∼47 kDa) could be a product of post-translational modification, alternative initiation, protein degradation or alternative mRNA splicing.

### Differential tegument extraction of SmStoLP-2 from adult worms

Extracts from schistosomula and adult worms were separated into soluble and insoluble fractions and Western blot analysis revealed SmStoLP-2 to be present in the insoluble fractions in both stages (Figure 2D). A higher molecular mass band can be seen in the insoluble fraction of schistosomula, similar to that observed in the recombinant protein (P). The two most prominent bands correspond closely to the monomeric and dimeric forms of the protein at 49 and 98 kDa.

To further characterize the distribution of SmStoLP-2 in *S. mansoni* tegument, differential extractions of tegument membrane proteins were analysed. Western blot using anti-rSmStoLP-2 serum, revealed that SmStoLP-2 was recovered in the first extraction fraction solubilized with urea/thiourea (Figure 2E), suggesting SmStoLP-2 to be firmly bound, although non-covalently, to the tegument membranes.

On the other hand, SmStoLP-2 displays a mitochondrial signal sequence, which, if functional, may target it to the tegumental mitochondria. To address this issue, we isolated the tegument and performed a differential fractionation, separating the membrane and mitochondrial fractions. The anti-rSmStoLP-2 antibody recognized the protein in both fractions (Figure 2F). Mitochondrial enrichment was ascertained using a Mitofusin-1 antibody, which only detected this protein in the mitochondria-enriched fraction.

### SmStoLP-2 is immunolocalized to the tegument of *S. mansoni*

**Figure 3:**
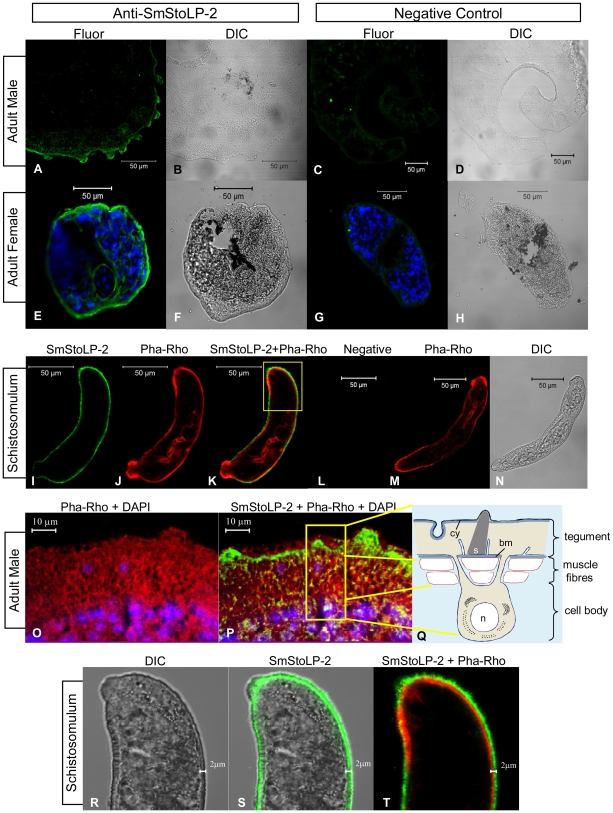
Immunolocalization of SmStoLP-2 in *S. mansoni* tegument. Fluorescence confocal microscopy images (Fluor) and corresponding differential interface contrast (DIC) images of male (A–D, O, and P), female adult worms (E–H), and schistosomulum (I–N and R–T) of *S. mansoni* are shown. Polyclonal anti-rSmStoLP-2 and secondary antibody coupled to Alexa 488 (green) were used for fluorescence detection of SmStoLP-2. Serum from naive rat was used as negative control for male (C), female (G) and schistosomulum (L). DAPI (blue) was used for nucleus localization (E, G, O, and P), and phalloidin rhodamine (red) was used for actin localization (J, K, M, O, P and T); (M) Diagram of the *S. mansoni* tegument and an associated cell body (not to scale). Cy: cytoskeleton; bm: basal membrane; n: nucleus; s: spine (extracted and modified from [43]).

Immunolocalization studies using rat serum raised against rSmStoLP-2 revealed through confocal fluorescence microscopy, that SmStoLP-2 is mainly expressed in the tegument of the adult *S. mansoni* male and female worms and seems to be expressed at lower levels in the muscle cells of male worms (Figure 3A and E). In an attempt to localize SmStoLP-2 in relation to the cytoskeletal tegument components, we used phalloidin-rhodamine as an actin marker. As shown in Figure 3P, there was some overlap staining on the muscle layers of adult male worms, revealed by the yellow signal. In contrast, the green band in the tegument, which corresponds to the main location of SmStoLP-2, did not seem to be co-localized with actin (Figure 3P). Additionally, the protein in male adult worms appears to be located more basally in the tegument, but it is interesting to note that the green band also seems to be running around and outside of their dorsal tubercles (Figure 3P).

Intact schistosomula stained with anti-rSmStoLP-2 and phalloidin-rhodamine suggested SmStoLP-2 to be external to the muscle layers in the tegument, as revealed by the green band running around and externally to the red band (Figure 3I–K, and R–T); additionally, the phalloidin-rhodamine internal labelling confirms that the parasites were well permeabilized (Figure 3J and M). No staining was observed in male and female sections or intact schistosomula incubated with naive rat serum (Figure 3C, G and L). Preliminary experiments indicate that, also in cercariae, SmStoLP-2 would be located in the evolving tegumental layer.

### Immunoglobulin isotype profile of schistosomiasis patients to rSmStoLP-2

**Figure 4:**
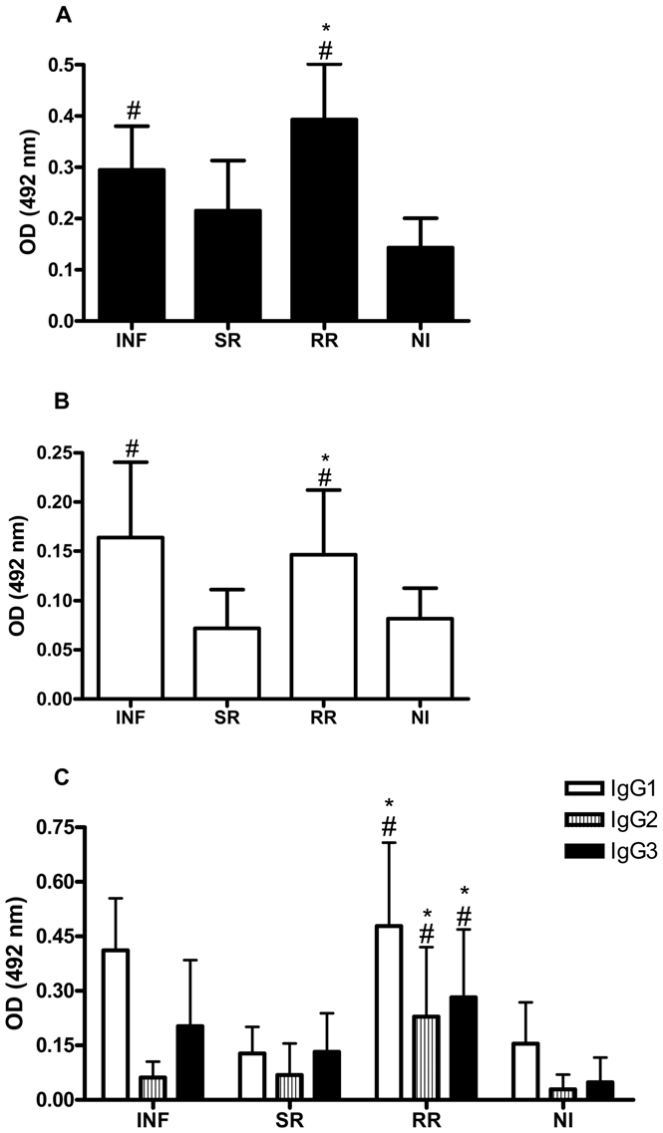
Isotype profile of sera from schistosomiasis patients reactive to rSmStoLP-2. Analysis of (A) IgG, (B) IgA and (C) IgG subclass antibody responses in sera of infected patients (INF), individuals susceptible to reinfection (SR), resistant to reinfection (RR) and non-infected individuals (NI). Results are expressed as means of individual measurements. Error bars indicate S.D. of the means. # Statistically significant as compared to the non-infected group (P<0.05). * Statistically significant as compared to the group susceptible to reinfection.

We evaluated by ELISA the specific reactivity of anti-SmStoLP-2 antibodies in sera of individuals with different status of resistance and susceptibility to *S. mansoni* reinfection. The sera of schistosomiasis patients, with the exception of the group susceptible to reinfection (SR), had significant levels of total anti-SmStoLP-2 IgG as compared to the non-infected group (Figure 4A). Furthermore, individuals from the group resistant to reinfection (RR) had increased levels of anti-SmStoLP-2 IgG when compared to individuals susceptible to reinfection (SR). Regarding IgA, statistically significant levels of antibodies to rSmStoLP-2 were observed in the INF and RR groups when compared to the NI group (Figure 4B). Once more, the RR group produced more anti-SmStoLP-2 IgA as compared to the SR individuals.

The IgG subclass profile of schistosomiasis patients was characterized predominantly by IgG1, IgG2 and IgG3 antibody responses to rSmStoLP-2. Individuals resistant to reinfection (RR) displayed at least a 2-fold higher level of IgG1, IgG2 and IgG3 anti-SmStoLP-2 antibodies as compared to those susceptible to reinfection (SR); these isotypes were also significantly higher when compared to the NI group (Figure 4C). Concerning IgG4, this antibody isotype was not detected in any of the groups studied.

### Inhibition of skin penetration

In an attempt to check if the anti-rSmStoLP-2 antibodies could impair penetration of cercariae and their survival afterwards, we performed a skin penetration inhibition assay. As shown in Figure S2A, the rat anti-rSmStoLP-2 serum inhibited cercarial skin penetration by 77%, as compared with 40% inhibition by serum from rats that received saline only (p = 0.002). Six weeks after the infection, we assessed the parasite load in the infected mice; data revealed that only 12% of the penetrating parasites matured to adult worms in the group in which cercariae were incubated with anti-rSmStoLP-2, while 42% matured in the group incubated with control serum (Figure S2B). In a typical infection in the murine model, usually the maturation rate is around 35–40% [42].

### Humoral immune response elicited by immunization with rSmStoLP-2

C57BL/6 mice were immunized with 3 doses of rSmStoLP-2 formulated with Freund's adjuvant and sera were analyzed by ELISA at 15, 30, 45, 60, 75 and 90 days for production of anti-SmStoLP-2 antibodies. Significant titers of specific anti-rSmStoLP-2 IgG antibodies were detected at all time points, showing a plateau after the third dose (data not shown). To determine the IgG isotype profile induced by immunization, specific IgG1 and IgG2a to rSmStoLP-2 were also analyzed. The levels of specific IgG1 and IgG2a and the IgG1/IgG2a ratio indicate that until the second dose there is a predominant Th2 response and after the third immunization there occurs a drift towards a more balanced or Th1-modulated immune response (Table 1).

**Table 1:** IgG1 and IgG2a immune profile induced by immunization of mice with rSmStoLP-2.

	Days^a^					
	15	30	45	60	75	90
IgG1^b^	0.88±0.33	1.26±0.05	1.31±0.04	1.28±0.05	1.25±0.09	1.32±0.06
IgG2a^b^	0.02±0.10	0.14±0.12	0.23±0.11	0.27±0.10	0.25±0.08	0.30±0.12
IgG1/IgG2a	31.7	9.0	5.6	4.7	4.9	4.3

a Days after the first immunization.

b Absorbance 492nm.

### Cytokine secretion induced by the recombinant protein

**Figure 5:**
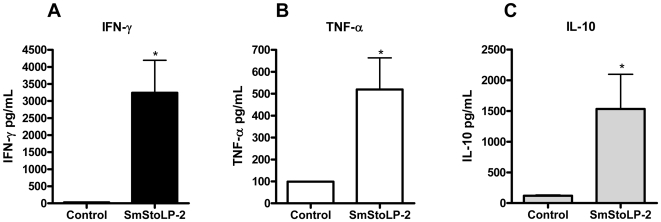
Cytokine profile of mice immunized with rSmStoLP-2. Ten days after the last immunization, splenocytes were isolated and assayed for (A) IFN-γ, (B) TNF-α and (C) IL-10 production in response to rSmStoLP-2 (25 µg/ml) or medium as control. The results are presented as mean ± S.D. for each group. Significant differences of secreted cytokines after rSmStoLP-2 stimulation or non-stimulated splenocytes are denoted by an asterisk (p<0.05).

In order to investigate the cytokine profile induced by the rSmStoLP-2 immunization regimen described above, we isolated splenocytes 10 days after the third immunization. Cytokine production (IFN-γ, TNF-α, IL-4 and IL-10) was measured in the culture supernatants from *in vitro* rSmStoLP-2-stimulated spleen cells of immunized mice. Statistically significant levels of IFN-γ, signature of Th1-type immune response, and TNF-α, a proinflammatory cytokine, were produced in the stimulated splenocytes from the rSmStoLP-2-immunized group as compared with the control (Figure 5A and B). Additionally, high levels of the modulatory cytokine, IL-10, were also observed (Figure 5C), and no secretion of IL-4, a Th2 cytokine, was detected (data not shown). These results indicate that immunization of mice with rSmStoLP-2 formulated with Freund's adjuvant induces a Th1-predominant immune response, with increased levels of IFN-γ, TNF-α and IL-10 and non-detectable levels of IL-4 secretion.

### Protection against challenge with cercariae

**Figure 6:**
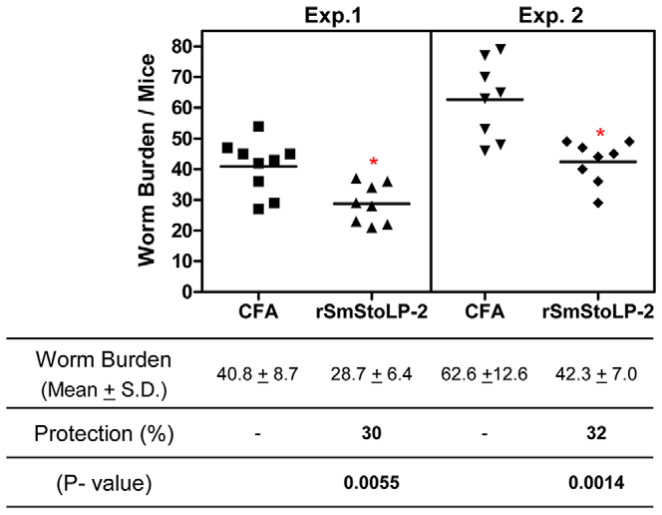
Scattergram of worm burden from two independent experiments of mice immunized with rSmStoLP-2 and challenged with live *S. mansoni* cercariae. Statistical analyses were performed with Student's t-test, *statistically significant (p<0.05) compared to control group (CFA/IFA).

In order to determine the protective potential of rSmStoLP-2, immunized mice were challenged with 100 cercariae. The worms were recovered by perfusion 6 weeks after challenge and results were expressed as the “mean worm burden” (mean ± S.D.) and are summarized in Figure 6. The animals immunized with rSmStoLP-2 in Freund's adjuvant showed 30 and 32% reduction in worm burden against challenge infection in two independent experiments when compared to the control group. Analysis of egg counts in the liver did not show a statistically significant reduction in oviposition.

## Discussion

In this report, we have identified SmStoLP-2 as a member of the stomatin super family, displaying several properties shared with other SLP-2 proteins and some unique features. The widespread distribution of the ‘conserved’ SPFH domain across life kingdoms has been taken as an indication of its ancient origin, suggesting the common ancestry and functional homology of all SPFH proteins [13]. However, in a recent review, it has been proposed that SPFH grouping has little phylogenetic support, probably due to convergent evolution of its members [41]. Independently of its origin, our phylogenetic analysis of the deduced SmStoLP-2 protein has grouped it together with human SLP-2, and SLP-2 from *Danio rerio* and *Xenopus tropicalis*, and at some distance from SLP-1, stomatin (band 7) and SLP-3. We can highlight the following primary sequence features: 1) SmStoLP-2 lacks an N-terminal hydrophobic domain, similar to other SLP-2 members [9]; 2) SmStoLP-2 and SjStoLP-2 are the unique members of SLP-2 family, which show putative sites for palmitoylation, a property that could enhance the hydrophobicity of proteins and contribute to their membrane association; 3) additionally, SmStoLP-2, like all SLP-2 proteins, seems to have a mitochondrial targeting peptide in the N-terminal region.

The recovery of SmStoLP-2 in the insoluble fraction of parasite extracts suggests that the protein could be membrane-associated. Furthermore, solubilization of SmStoLP-2 from tegument membranes after the treatment with the chaotropic agents, urea and thiourea, indicates that it should be non-covalently bound to the tegument. It is interesting to note that SmStoLP-2 was not identified on Braschi's proteomic study [30], which could be explained based on the differences in sensitivity of the two methods used to detected the protein (mass spectrometry and Western blot). Moreover, albeit not with a quantitative analysis, the fact that SmStoLP-2 protein is present in the free-living (freshwater) cercariae and miracidia, as well as in the egg stage, suggests that the protein has other function(s), not exclusively associated with the tegument, which are common to both free-living and parasitic stages.

It has been recently proposed that HuSLP-2 may interact with actin (a cytoskeletal constituent), vav (a small GTPase that regulates cytoskeleton reorganization) and Nck (an adaptor protein that links transmembrane and scaffolding molecules to the cytoskeleton) [23]. The confocal immunofluorescence images of the parasites confirm the SmStoLP-2 tegument localization and suggest some weak co-localization with actin, only on muscle layers of adult male worms. In addition, SmStoLP-2 seems to be located externally to the muscle layers in the 7-day-old schistosomulum.

Data from the HuSLP-2 suggests that there are at least two cellular pools of this protein: one associated with the plasma membrane and the other with mitochondria [9],[11],[24]. It is important to note that the tegumental cytoplasmic layer lying under the surface membranes, contains small mitochondria [43]. However, confocal immunofluorescence microscopy does not have sufficient resolution to address this question with confidence. Our results on the differential fractionation of tegument extracts analyzed by Western blot addressed this issue and strongly suggested that SmStoLP-2 also displays a dual targeting, one associated to the tegument membrane and one to the mitochondria.

As a consequence of the studies in the attenuated cercaria vaccine model, the schistosomula is believed to be the target of protective immunity [42]. Given the tegument localization of SmStoLP-2 in the schistosomula suggested by our immunolocalization results, this molecule should be accessible as an immune target. In individuals putatively resistant to reinfection (RR), the antibody response mounted against SmStoLP-2, consisted mainly of the cytophilic antibodies IgG1 and IgG3, which have opsonization properties, cell dependent cytotoxicity, and the ability to activate the classical complement pathway, functions which could be involved in the resistance to *S. mansoni* reinfection. Elevated levels of IgG1, IgG2 and IgG3 have been linked to the human resistant status for several vaccine candidates, such as, Sm23, Sm28, Sm14-FABP, Sm29 and TSP-2 [32],[44],[45]. Concerning IgA levels, investigators have associated the increased levels of this isotype with resistance to reinfection stimulated by Sm28GST antigen [46],[47], which parallels our results, where high levels of IgA antibodies to rSmStoLP-2 were observed in patients which are resistant to reinfection (RR).

Although no function has been ascribed for SmStoLP-2, our finding that anti-rSmStoLP-2 antibodies inhibits cercariae skin penetration and migration, suggests that the molecule may have an important role in larval host entry and in migration through the tissues until the lungs before reaching the portal hepatic system. This finding provides further support for testing SmStoLP-2 as a vaccine candidate against murine schistosomiasis.

In the murine model, rSmStoLP-2 induced high levels of anti-rSmStoLP-2 IgG after the second immunization and showed a reduced IgG1/IgG2a ratio at 45 days after the first immunization. Additionally, we confirmed by cytokine analysis that rSmStoLP-2 immunization elicited a Th1-predominant type of immune response characterized by production of high levels of IFN-γ and no detection of IL-4. To determine if rSmStoLP-2 conferred protection against *S. mansoni* infection, immunized mice were challenged with cercaria and worm burden analyzed. Immunization with rSmStoLP-2 induced a 30-32% worm burden reduction.

A primary obstacle in the research and development of a schistosomiasis vaccine is a lack of understanding of what type of immune response should be induced. In the irradiated cercariae vaccination model, protection can be either based on a Th1, a Th2, or a mixed Th1/Th2 immune response [48]. However, in the case of recombinant proteins, Th1 inducing antigens have been described to induce protection against infection in the mouse model [36], [49]–[53]. The role of IFN-γ in the protective immunity to schistosomiasis is well described in mice exposed to the irradiated vaccine and there is compelling evidence that immune elimination of challenge parasites occurs in the lungs. Since IFN-γ is likely to be required to activate pulmonary macrophages which may mediate the protective response [54], we could expect that similar mechanisms would be involved in rSmStoLP-2 protective immunity.

Recently, HuSLP-2 has been proposed as a potentially useful target for immunotherapy in humans, since it modulates effector T cell responses [24]. Thus, down-regulation of HuSLP-2 expression could be valuable in the course of autoimmune disease treatment, since it decreases T cell reactivity; alternatively, enhancement of HuSLP-2 expression could be explored in vaccine development, since it would increase T cell responsiveness [24]. Given that orthologs often, but not always, have the same function, it is still unclear what is the function of SmStoLP-2; it could have an immunomodulatory role like its human ortholog [24] or could acquire a different function on the parasite-host interface, like providing structural scaffolding for the tegument or supporting the traffic of vesicles to the surface plasma membrane [9], organizing the peripheral cytoskeleton and assembly of multichain receptors, such as ion channels [11],[55], or even mechanosensation receptors [18],[20]. Further investigations of SmStoLP-2 will be valuable in understanding how the tegument functions as the parasite-host interface.

A critical issue in vaccine design is the use of an appropriate adjuvant and/or delivery system to induce the suitable immune response. Experiments are underway investigating rSmStoLP-2 with different adjuvant formulations, which would be suitable for use in humans. In conclusion, our study showed that SmStoLP-2 is a novel tegument protein, being recognized by different subclasses of antibodies in patients resistant and susceptible to reinfection, and in the light of data obtained from murine studies, protective properties against schistosomiasis were revealed. We believe that an ideal vaccine may require the combination of quite a few antigen targets to induce an effective protection against the parasite, and SmStoLP-2 could contribute to reach this goal.

## Supporting Information

Figure S1 Phylogenetic analysis performed with protein sequences showing that SmStoLP-2 is part of the stomatin family. The sequences accession numbers are: *X. tropicalis* hypothetical (GenBank accession no. NP_001004808.1), human prohibitin (AAP36079), *S. mansoni* putative prohibitin-1 (GeneDB accession no. Smp_075210.2), Yeast prohibitin 1 (NP_011648), *S. mansoni* putative prohibitin-2 (Smp_075940), Yeast prohibitin 2 (NP_011747), human flotillin-1 (AAP36527), *S. mansoni* putative flotillin-1 (Smp_016200.1), human flotillin- 2 (NP_004466), *S. mansoni* putative flotillin-2 (Smp_033970), *Escherichia coli* HflK (NP_458799), *E. coli* HflC (NP_418596). (Accession numbers of the other members are cited in the legend of Figure 1).
(0.01 MB PDF)

Figure S2 Inhibition of cercariae skin penetration by rat anti-SmStoLP-2 antiserum. For these studies, 100 *S. mansoni* cercariae were applied in pond water for percutaneous infection and the number of non-penetrating parasites were counted. The percentage inhibition resulting from either rat-anti-SmStoLP-2 antiserum or antiserum obtained from control rats immunized with saline is expressed as the mean ± S.D. of one representative of three independent experiments.
(0.01 MB PDF)

Table S1 Study population.
(0.01 MB PDF)
