# Supplementary figures and images for: Schistosoma mansoni Stomatin Like Protein-2 Is Located in the Tegument and Induces Partial Protection against Challenge Infection
Source: PLoS Negl Trop Dis. 2010 Feb 9;4(2):e597. doi: 10.1371/journal.pntd.0000597 (PMC2817717; doi:10.1371/journal.pntd.0000597)

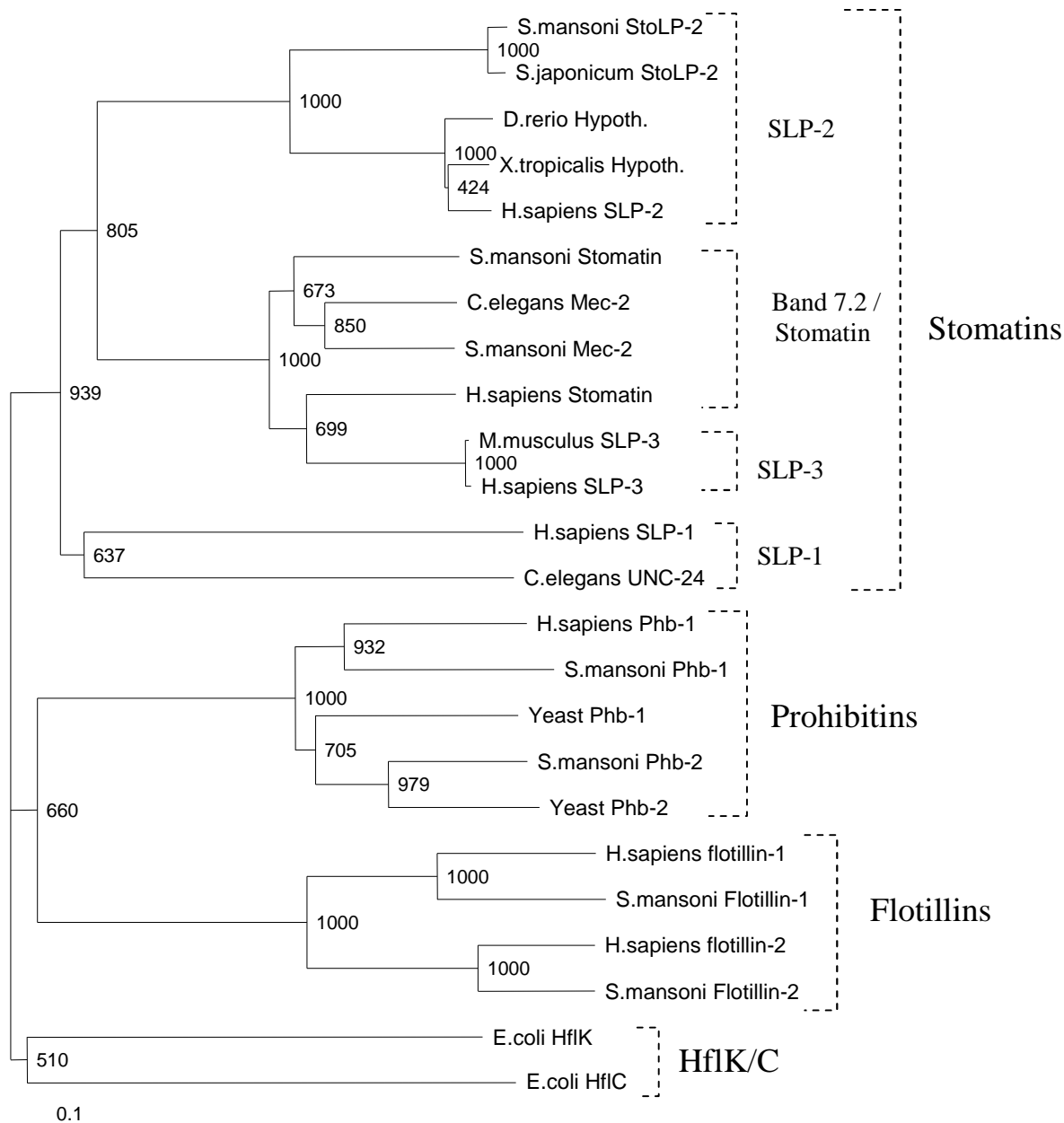

Supplement: Figure S1 — Phylogenetic analysis performed with protein sequences showing that SmStoLP-2 is part of the stomatin family. The sequences accession numbers are: X. tropicalis hypothetical (GenBank accession no. NP_001004808.1), human prohibitin (AAP36079), S. mansoni putative prohibitin-1 (GeneDB accession no. Smp_075210.2), Yeast prohibitin 1 (NP_011648), S. mansoni putative prohibitin-2 (Smp_075940), Yeast prohibitin 2 (NP_011747), human flotillin-1 (AAP36527), S. mansoni putative flotillin-1 (Smp_016200.1), human flotillin- 2 (NP_004466), S. mansoni putative flotillin-2 (Smp_033970), Escherichia coli HflK (NP_458799), E. coli HflC (NP_418596). (Accession numbers of the other members are cited in the legend of Figure 1). (0.01 MB PDF) [file pntd.0000597.s001.pdf]

**A**

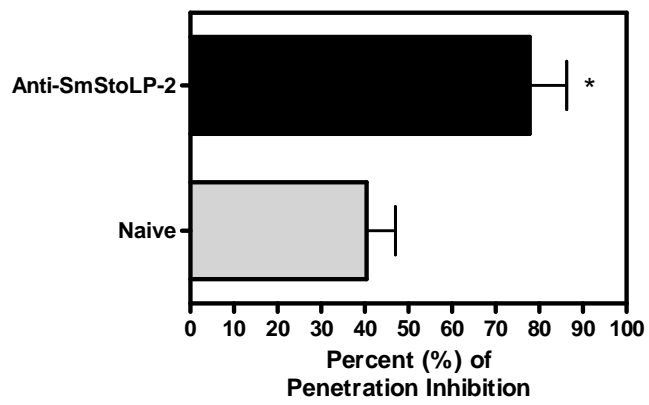

**B**

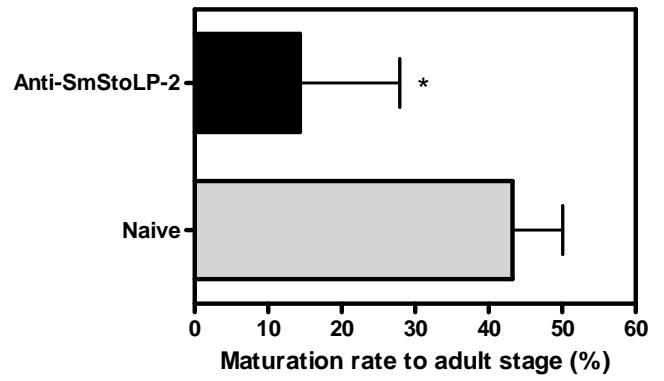

Supplement: Figure S2 — Inhibition of cercariae skin penetration by rat anti-SmStoLP-2 antiserum. For these studies, 100 S. mansoni cercariae were applied in pond water for percutaneous infection and the number of non-penetrating parasites were counted. The percentage inhibition resulting from either rat-anti-SmStoLP-2 antiserum or antiserum obtained from control rats immunized with saline is expressed as the mean ± S.D. of one representative of three independent experiments. (0.01 MB PDF) [file pntd.0000597.s002.pdf]
