# Supplementary material for: Schistosoma mansoni Stomatin Like Protein-2 Is Located in the Tegument and Induces Partial Protection against Challenge Infection
Source: PLoS Negl Trop Dis. 2010 Feb 9;4(2):e597. doi: 10.1371/journal.pntd.0000597 (PMC2817717; doi:10.1371/journal.pntd.0000597)
Supplement: Table S1 — Study population. (0.01 MB PDF) [file pntd.0000597.s003.pdf]

**Table S1 -** Study population.

| Group | Infection status | Description | Age (mean ± SD) | Sex (M/F) |
|---|---|---|---|---|
| Infected (I) *n* = 11 | Infected with *S. mansoni* | Individuals living in an endemic area for schistosomiasis with stool positive examinations | 19.2 ± 10.9 | 6/5 |
| Susceptible to reinfection (SR) *n* = 12 | Infected with *S. mansoni* | Individuals living in an endemic area for schistosomiasis that present stool positive examination after praziquantel treatment | 22.8 ± 12.1 | 7/5 |
| Resistant to reinfection (RR) *n* = 11 | Not infected | Individuals living in an endemic area for schistosomiasis that although having water contact, present stool negative examination after praziquantel treatment | 20.8 ± 8.8 | 5/6 |
| Not infected (NI) *n* = 9 | Not infected | Individuals living out of endemic area for schistosomiasis that never presented this disease in their lives | 28.3 ± 4.5 | 4/5 |
